# Mid-regional pro-adrenomedullin is a novel biomarker for arterial stiffness as the criterion for vascular failure in a cross-sectional study

**DOI:** 10.1038/s41598-020-79525-2

**Published:** 2021-01-11

**Authors:** Teruhide Koyama, Nagato Kuriyama, Yosuke Suzuki, Satoshi Saito, Ryota Tanaka, Motoshi Iwao, Megumu Tanaka, Takakuni Maki, Hiroki Itoh, Masafumi Ihara, Takayuki Shindo, Ritei Uehara

**Affiliations:** 1 Department of Epidemiology for Community Health and Medicine, Kyoto Prefectural University of Medicine, Kyoto, Japan; 2 Department of Medication Use Analysis and Clinical Research, Meiji Pharmaceutical University, Kiyose, Japan; 3 Department of Clinical Pharmacy, Oita University Hospital, Oita, Japan; 4 Department of Neurology, National Cerebral and Cardiovascular Center, Suita, Japan; 5 Department of Cardiovascular Research, Shinshu University Graduate School of Medicine, Matsumoto, Japan; 6 Department of Life Innovation, Institute for Biomedical Sciences, Interdisciplinary Cluster for Cutting Edge Research, Shinshu University, Matsumoto, Japan; 7 Department of Neurology, Graduate School of Medicine, Kyoto University, Kyoto, Japan

**Keywords:** Cardiovascular biology, Cardiovascular diseases, Vascular diseases

## Abstract

We investigated the potential of mid-regional pro-adrenomedullin (MR-proADM) for use as a novel biomarker for arterial stiffness as the criterion for vascular failure and cardiometabolic disease (obesity, hypertension, dyslipidemia, diabetes, and metabolic syndrome) compared with high-sensitivity C-reactive protein (hsCRP). Overall, 2169 individuals (702 men and 1467 women) were enrolled. Multiple regression analysis was performed to assess the association of MR-proADM and hsCRP with brachial-ankle pulse wave velocity (baPWV), adjusting for other variables. The diagnostic performance (accuracy) of MR-proADM with regard to the index of vascular failure was tested with the help of receiver operating characteristic curve analysis in the models. MR-proADM was significantly higher in participants with vascular failure, as defined by baPWV and/or its risk factors (obesity, hypertension, dyslipidemia, diabetes, and metabolic syndrome), than in control groups. Independent of cardiovascular risk factors (age, drinking, smoking, body mass index, systolic blood pressure, lipid and glycol metabolism), MR-proADM was significantly associated with baPWV, and MR-proADM showed higher areas under the curve of baPWV than hsCRP showed. MR-proADM is more suitable for the diagnosis of higher arterial stiffness as the criterion for vascular failure than hsCRP. Because vascular assessment is important to mitigate the most significant modifiable cardiovascular risk factors, MR-proADM may be useful as a novel biomarker on routine blood examination.

## Introduction

Vascular failure is defined as the integration of endothelial dysfunction, smooth muscle dysfunction, and metabolic abnormalities of the vessel wall^1^. In 2018, new physiological diagnostic criteria for vascular failure were proposed by the Physiological Diagnosis Criteria for Vascular Failure Committee of the Japan Society for Vascular Failure, according to the target vascular layers and areas, assessed by endothelial function and arterial stiffness (a marker integrating medial layer function) using universally available diagnostic tools^2^. Endothelial function can be measured by flow-mediated vasodilation (FMD) in the brachial artery and reactive hyperemia-peripheral arterial tonometry (RH-PAT) in the fingertip. Arterial stiffness, which is reflected by medial layer function, can be assessed by pulse wave velocity (PWV) and cardio-ankle vascular index (CAVI)^2^. However, no blood biomarker is known to be diagnostic of vascular failure.

Adrenomedullin (ADM) is a vasoactive peptide identified in human pheochromocytoma^3^. Although ADM is secreted from various organs and tissues, it is produced mainly by vascular endothelial cells and serves a number of physiological functions^4,5^. Of note, plasma ADM levels are elevated in patients with hypertension, congestive heart failure or myocardial infarction^6,7^, renal diseases^8^, diabetes mellitus^9^, the acute phase of stroke^10^, septic shock^11^, arterial stiffness assessed by PWV^12^, and the magnitude of the elevation is in proportion to the severity of the disease involving vascular damage.

However, reliable measurement of ADM levels is challenging due to its short half-life of 22 min^13^. The precursor molecule of ADM, mid-regional pro-ADM (MR-proADM), has higher stability, which allows it to be reliably measured as a surrogate biomarker for ADM^14^. In sum, the hypothesis holds that MR-proADM, like ADM, is associated with cardiometabolic diseases and arterial stiffness. Although the evidence for a classical cardiovascular biomarker, High-sensitivity C-reactive protein (hsCRP), in disease etiology has been initially promising, the evidence for a causal role in humans remains limited^15^. This study aimed the potential of MR-proADM for use as a novel biomarker for arterial stiffness assessed by PWV as the criterion for vascular failure and cardiometabolic disease (obesity, hypertension, dyslipidemia, diabetes, and metabolic syndrome) compared with hsCRP.

## Results

**Figure 1:**
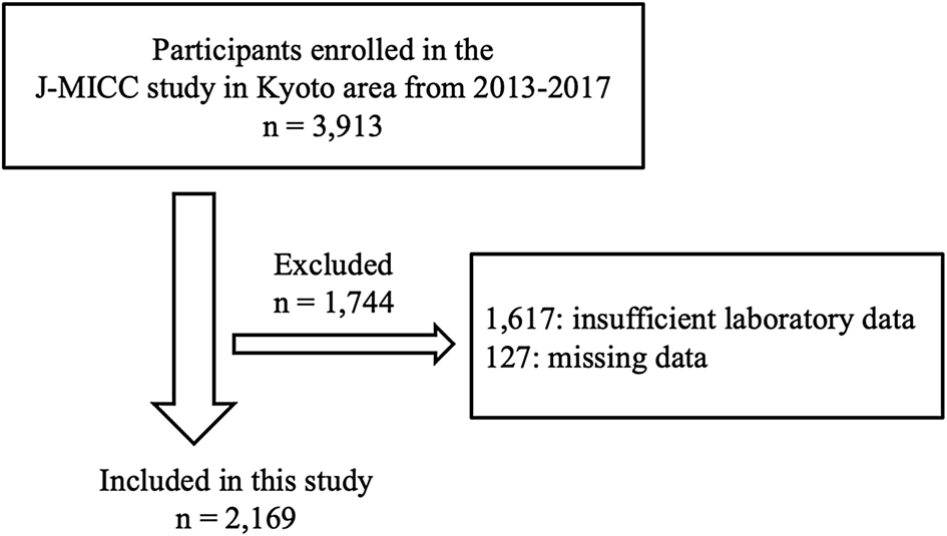
Flow chart of the study participants.

Figure 1 shows the study participant flow chart. Table 1 showed the anthropometric measurements and participant characteristics in this study. The mean age was 59.8 ± 10.3 years in men and 57.3 ± 9.89 years in women. The MR-proADM level was significantly different between men and women, 0.468 ± 0.100 nmol/L in men versus 0.412 ± 0.082 nmol/L in women (*P* < 0.001).

**Table 1:** Participant characteristics at baseline according to sex.

	Men (n = 702)	Women (n = 1467)	All (n = 2169)
	Mean ± SD	Mean ± SD	Mean ± SD
Age (years)	59.8 ± 10.3	57.3 ± 9.89	58.1 ± 10.1
Body mass index (kg/m^2^)	23.5 ± 2.99	21.6 ± 3.15	22.2 ± 3.22
Systolic blood pressure (mmHg)	135 ± 17.3	125 ± 19.3	128 ± 19.1
Diastolic blood pressure (mmHg)	82.3 ± 10.5	76.4 ± 11.1	78.3 ± 11.2
Triglycerides (mg/dL)	124 ± 86.7	90.3 ± 63.9	101 ± 73.8
Total cholesterol (mg/dL)	204 ± 32.0	222 ± 37.4	216 ± 36.8
Low-density lipoprotein cholesterol (mg/dL)	121 ± 29.5	127 ± 31.7	125 ± 31.1
High-density lipoprotein cholesterol (mg/dL)	60.1 ± 14.4	73.9 ± 16.5	69.5 ± 17.1
Blood sugar (mg/dL)	94.9 ± 15.9	88.8 ± 12.8	90.8 ± 14.2
A glycated hemoglobin (%)	5.66 ± 0.55	5.59 ± 0.40	5.59 ± 0.46
Waist circumstance (cm)	85.1 ± 8.20	78.8 ± 8.91	80.8 ± 9.18
Brachial-ankle pulse wave velocity (cm/s)	1496 ± 334	1359 ± 316	1403 ± 329
Mid-regional pro-adrenomedullin (nmol/L)	0.468 ± 0.100	0.412 ± 0.082	0.430 ± 0.092
High-sensitivity C-reactive protein	0.055 ± 0.069	0.044 ± 0.064	0.047 ± 0.066

	n (%)	n (%)	n (%)
**Alcohol drinking**			
Current	524 (74.6)	745 (50.8)	1269 (58.5)
Former	11 (1.6)	18 (1.2)	29 (1.3)
Never	167 (23.8)	704 (48.0)	871 (40.2)
**Smoking**			
Current	125 (17.8)	68 (4.6)	193 (8.9)
Former	338 (48.1)	184 (12.5)	522 (24.1)
Never	239 (34.0)	1215 (82.8)	1454 (67.0)
**Drug treatment**			
Hypertension	181 (25.8)	174 (11.9)	355 (16.4)
Dyslipidemia	118 (16.8)	227 (15.5)	345 (15.9)
Diabetes	51 (7.3)	26 (1.8)	77 (3.6)

The means of MR-proADM, according to cardiometabolic disease and sex, are shown in Table 2. In both sexes, the mean MR-proADM levels were significantly higher in participants with vascular failure, as defined by baPWV and/or risk factors (obesity, hypertension, dyslipidemia, diabetes, and MS), than in control groups. Supplemental Table S1 shows the means of MR-proADM according to the sum of four cardiometabolic diseases (obesity, hypertension, dyslipidemia, and diabetes). In both sexes, the means of MR-proADM value tended to increase as the number of cardiometabolic diseases increased.

**Table 2:** The means of mid-regional pro-adrenomedullin according to cardiometabolic disease and brachial-ankle pulse wave velocity.

		%	Men	p-value		%	Women	p-value
			Mean ± SD				Mean ± SD	
Body Mass Index < 25	n = 500	71.2	0.460 ± 0.096	**0.001**	n = 1263	86.1	0.404 ± 0.077	** < 0.001**
Body Mass Index ≥ 25	n = 202	28.8	0.487 ± 0.106		n = 204	13.9	0.466 ± 0.093	
Hypertension (−)	n = 342	48.7	0.438 ± 0.077	** < 0.001**	n = 1038	70.8	0.396 ± 0.072	** < 0.001**
Hypertension (+)	n = 360	51.3	0.496 ± 0.110		n = 429	29.2	0.451 ± 0.093	
Dyslipidemia (−)	n = 387	55.1	0.459 ± 0.099	**0.007**	n = 803	32.2	0.400 ± 0.080	**0.001**
Dyslipidemia (+)	n = 315	44.9	0.479 ± 0.099		n = 664	67.8	0.428 ± 0.082	
Diabetes (−)	n = 619	88.2	0.463 ± 0.097	**0.001**	n = 1413	96.3	0.411 ± 0.081	** < 0.001**
Diabetes (+)	n = 83	11.8	0.502 ± 0.110		n = 54	3.7	0.464 ± 0.109	
Metabolic syndrome (−)	n = 485	69.1	0.456 ± 0.093	** < 0.001**	n = 1360	92.7	0.406 ± 0.078	** < 0.001**
Metabolic syndrome (+)	n = 217	30.9	0.497 ± 0.108		n = 107	7.3	0.489 ± 0.098	
Brachial-ankle pulse wave velocity < 1400	n = 331	47.2	0.431 ± 0.082	** < 0.001**	n = 908	61.9	0.394 ± 0.073	** < 0.001**
Brachial-ankle pulse wave velocity ≥ 1400	n = 370	52.8	0.501 ± 0.103		n = 559	38.1	0.443 ± 0.088	
Brachial-ankle pulse wave velocity < 1800	n = 581	82.8	0.457 ± 0.096	** < 0.001**	n = 1338	91.2	0.407 ± 0.077	** < 0.001**
Brachial-ankle pulse wave velocity ≥ 1800	n = 121	17.2	0.517 ± 0.104		n = 129	8.8	0.470 ± 0.108	

Category differences are analyzed by Welch’s t-tests.

Bold style represents p < 0.05.

Correlations between MR-proADM and the cardiometabolic parameters are shown in Table 3. Although most of the cardiometabolic parameters were significantly associated with MR-proADM, among them age and baPWV showed higher correlation coefficients for both sexes.

**Table 3:** Correlations between mid-regional pro-adrenomedullin and cardiometabolic parameters.

	Men (n = 702)		Women (n = 1467)	
	Coefficient	*p*-value	Coefficient	*p*-value
Age (years)	0.510	** < 0.001**	0.430	** < 0.001**
Body Mass Index	0.123	**0.001**	0.287	**< 0.001**
Systolic blood pressure	0.230	** < 0.001**	0.324	**< 0.001**
Diastolic blood pressure	0.058	0.123	0.220	**< 0.001**
Triglycerides	0.233	** < 0.001**	0.245	**< 0.001**
Total cholesterol	− 0.021	0.583	0.102	**< 0.001**
Low-density lipoprotein cholesterol	− 0.084	**0.026**	0.112	**< 0.001**
High-density lipoprotein cholesterol	− 0.025	0.505	− 0.105	**< 0.001**
Blood sugar	0.112	**0.003**	0.164	**< 0.001**
A glycated hemoglobin	0.144	** < 0.001**	0.166	**< 0.001**
Waist circumstance	0.231	** < 0.001**	0.343	**< 0.001**
Brachial-ankle pulse wave velocity	0.426	** < 0.001**	0.385	**< 0.001**
High-sensitivity C-reactive protein	0.298	** < 0.001**	0.274	**< 0.001**

Correlations are analyzed by Spearman’s rank correlation analysis.

Bold style represents p < 0.05.

In multivariable logistic regression adjusting for known cardiovascular risk factors (age, drinking, smoking, BMI, SBP, lipid and glycol metabolism), MR-proADM was significantly associated with baPWV (men: beta = 0.111, *p* < 0.001, women: beta = 0.077, *p* < 0.001), and that most cardiometabolic parameters. However, hsCRP, the classical cardiovascular risk biomarker, was not significantly associated with baPWV (Table 4).

**Table 4:** Multivariate analysis of association with brachial-ankle pulse wave velocity.

	Men (n = 702)		Women (n = 1467)	
	beta	*p*-value	beta	*p*-value
Mid-regional pro-adrenomedullin	0.111	** < 0.001**	0.077	** < 0.001**
High-sensitivity C-reactive protein	0.013	0.617	0.020	0.236
Age	0.335	** < 0.001**	0.373	** < 0.001**
Body Mass Index	− 0.171	** < 0.001**	− 0.121	** < 0.001**
Systolic blood pressure	0.477	** < 0.001**	0.520	** < 0.001**
Triglycerides	0.072	**0.009**	0.047	**0.009**
High-density lipoprotein cholesterol	0.024	0.417	− 0.020	0.257
Low-density lipoprotein cholesterol	− 0.035	0.174	0.007	0.660
A glycated hemoglobin	0.089	**0.001**	0.035	**0.045**

Adjusted for age, drinking and smoking status.

Analysis were performed by multiple regression analysis.

Bold style represents p < 0.05.

**Figure 2:**
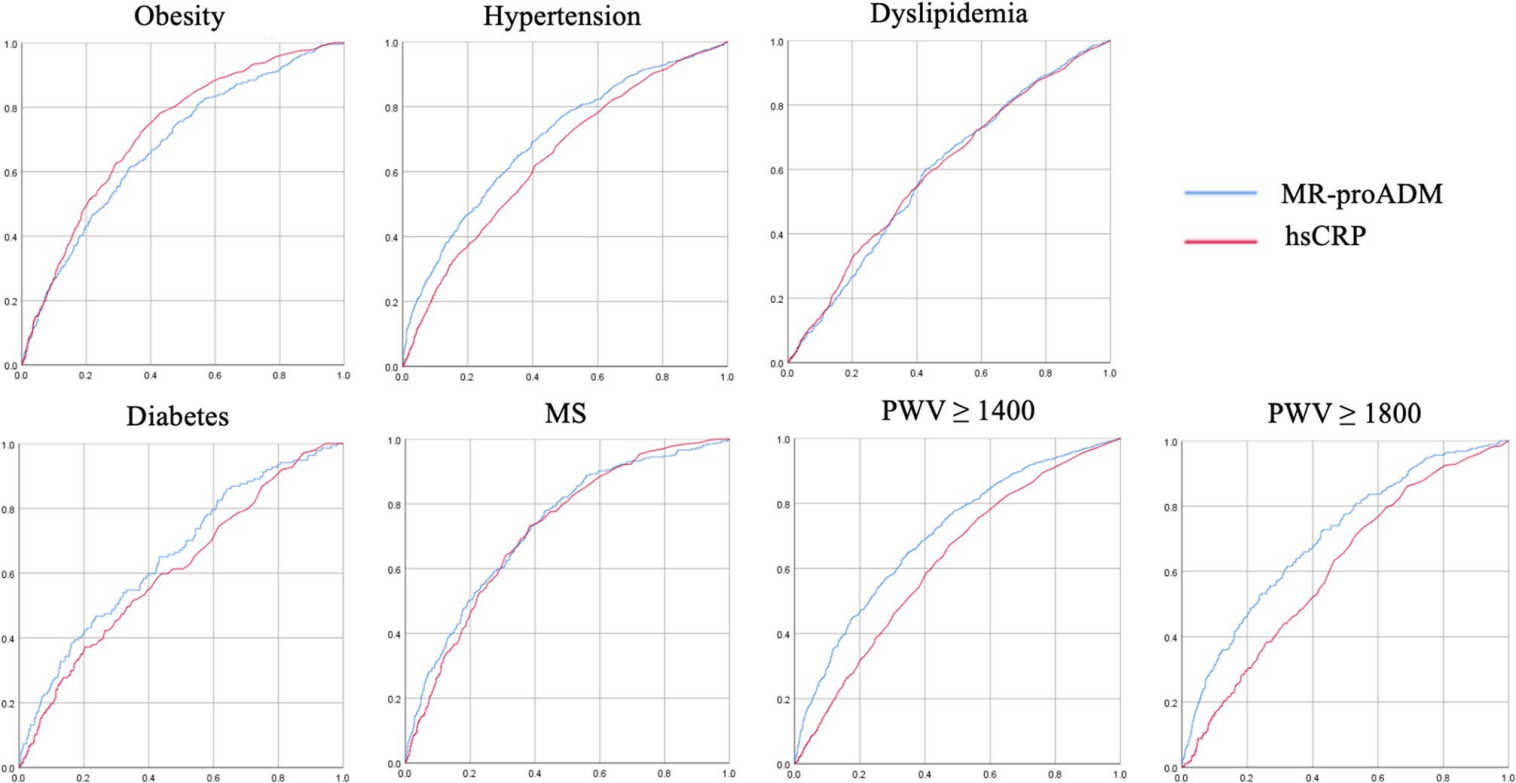
Receiver operating characteristic curve analysis classified cardiometabolic disease and arterial stiffness by MR-proADM and hsCRP.

Since we demonstrated that MR-proADM might prove to be a significant biomarker for vascular failure, we calculated the receiver operating characteristic (ROC) curve (Fig. 2). ROC analysis showed that MR-proADM had both high sensitivity and specificity in predicting the baPWV, which is the cutoff value for vascular failure than hsCRP. The areas under the curve (AUC) of the model with MR-proADM and hsCRP were significantly associated with all variables (data not shown). Moreover, MR-proADM showed higher AUC of baPWV, which defined vascular failure, than did hsCRP.

## Discussion

The main findings of the present study can be summarized as follows: first, MR-proADM was significantly higher in participants with vascular failure, as defined by baPWV and/or its risk factors (obesity, hypertension, dyslipidemia, diabetes, and MS), than in control groups. Second, independent of cardiovascular risk factors (age, drinking, smoking, BMI, SBP, lipid, and glycol metabolism), MR-proADM was significantly associated with baPWV, and MR-proADM showed higher AUC of baPWV than hsCRP showed. To our knowledge, this study is the first to report MR-proADM as a more suitable biomarker for vascular failure as defined by baPWV and its risk factors than hsCRP.

Our results showed that MR-proADM levels were significantly higher in participants with obesity, hypertension, dyslipidemia, diabetes, and MS than in control participants, and that most cardiometabolic parameters were significantly associated with MR-proADM. In agreement with many previous studies, our study confirmed that MR-proADM was associated with cardiovascular risk factors^16–20^.

ADM has also been shown also to have anti-inflammatory and anti-oxidative properties and to limit the arterial intimal hyperplasia, a response to protect organs from damage^21–24^. Indeed, the relationship between ADM signal and vascular integrity has been investigated in many experimental^4,25–27^ and some epidemiological studies^28–30^. As a consequence, the increase of ADM levels found in these pathological disorders affecting the vascular system may be seen as a counter-regulatory (compensatory) mechanism for vascular damage. In sum, MR-proADM is associated with cardiometabolic parameters and is an independent predictor for cardiovascular events in patients^31–33^.

In this study, we used only baPWV as a diagnostic of vascular failure. However, physiological diagnostic criteria of vascular failure were based on vascular functional parameters: endothelial function and arterial stiffness. Endothelial function was measured by FMD and/or RH-PAT^2^. One study, the Gutenberg Health Study, showed that MR-proADM was significantly associated with RH-PAT after adjusting for cardiovascular risk; however, results have been inconsistent for FMD. In brief, MR-proADM was not significantly associated with FMD after adjusting for cardiovascular risk factors: age, sex, BMI, diabetes, current smoking, logarithmically transformed pulse pressure, dyslipidemia, and a positive family history of myocardial infarction^34^. The relationship between ADM and endothelial function from laboratory data is well known^4^ and it was required further study of endothelial function and ADM using a large number of human samples.

ADM has potential for therapeutic applications, but its use is limited by its short half-life in the bloodstream. On the other hand, MR-proADM has higher stability^14^. In this study, MR-proADM plasma concentrations were measured by an automated KRYPTOR analyzer, which is used commercially in some countries^14^. Using UPLC-MS/MS, we recently established a sensitive method for determining plasma MR-proADM concentration^35^. Our novel UPLC-MS/MS assay for determining MR-proADM concentration can be used in the clinical setting and may have better selectivity than the immunoassay method. This means that MR-proADM can be measured on routine examination.

The limitations of our study include its cross-sectional design and the inclusion of only Japanese participants. Recall bias is unavoidable because we use the self-administered questionnaires study. Therefore, the value of the self-reported lifestyle factor may not have been accurate enough. If present, however, such information is non-specific and can be prevented by the large sample size. The strength of this study is the inclusion of a large number of Japanese participants, which prevented sample bias. Another limitation is that the mean cut-off value for MR-proADM is yet to be established, and we were not able to categorize the MR-proADM of the participants. On the other hand, MR-pro ADM measurement in many Japanese is novel and study strength.

Our results show that MR-proADM is more suitable for the diagnosis of higher arterial stiffness as the criterion for vascular failure than hsCRP. Because vascular assessment is important to mitigate the most significant modifiable cardiovascular risk factors, MR-proADM may be useful as a novel biomarker on routine blood examination.

## Methods

### Participants

The Japan Multi-Institutional Collaborative Cohort Study, was launched in 2005 to investigate gene–environmental interactions in lifestyle-related diseases^36,37^. This study included individuals who were enrolled in the Japan Multi-Institutional Collaborative Cohort (J-MICC) Study second survey in the Kyoto area from 2013 to 2017. A total of 3913 participants were eligible for analyses. All subjects underwent a routine health checkup. Among these 3913 participants, 1744 were excluded, 1617 due to insufficient laboratory data and 127 due to the absence of other data. After these exclusions, 2169 individuals (702 men and 1467 women) were eligible for analyses. Inclusion criteria was all participants with no missing variables.

The study was approved by the Institutional Ethics Committee of Kyoto Prefectural University of Medicine (approval number: RBMR-E-36-8 at 2013) and was conducted in accordance with the principles of the Declaration of Helsinki. All participants provided written informed consent before participation.

### Clinical and biochemical analysis

The following lifestyle and medical information obtained through self-administered questionnaires which used in J-MICC Study were evaluated: alcohol consumption and smoking status (current, former, never) and current medications. In addition, anthropometric data obtained from the health check-ups were collected^38^. Waist circumstance (WC) was measured at the umbilical level in minimal respiration in a standing position. Body mass index (BMI) was calculated as weight divided by the square of height (kg/m^2^). The cut-off point for obesity was set at a BMI of 25 kg/m^2^ according to the definition of obesity in Japan^39^.

Anamnesis and medication history were assessed using self-administered questionnaires^38^. Hypertension was defined as a systolic/diastolic blood pressure (SBP/DBP) ≥ 140/90 mmHg and/or current use of medication for hypertension. Dyslipidemia was defined as low-density lipoprotein cholesterol (LDL-C) ≥ 140 mg/dL and/or high-density lipoprotein cholesterol (HDL-C) < 40 mg/dL and/or current use of medication for dyslipidemia. Diabetes was defined as a glycated hemoglobin (HbA1c) level ≥ 6.5% and/or blood sugar (BS) ≥ 126 and/or current use of medication for diabetes. The Japanese criteria of metabolic syndrome (MS) was defined as follows: if a man has a WC ≥ 85 cm (in the case of a woman, ≥ 90 cm) in addition to two or more of the following: lipid abnormality: high triglyceride level (≥ 150 mg/dL), and/or HDL-cholesterol level (≤ 40 mg/dL), or the use of lipid-modifying drugs; elevated blood pressure: SBP ≥ 130 mmHg and/or DBP ≥ 85 mmHg, or the use of antihypertensive drugs; and elevated blood glucose: HbA1c 5.6% and/or BS ≥ 100, or the use of drugs for diabetes^40^.

The brachial-ankle pulse wave velocity (baPWV) used to evaluate arterial stiffness was measured with a volume-plethysmographic apparatus (BP-203RPE II form PWV/ABI, Omron Healthcare Co. Ltd., Kyoto, Japan). We simultaneously measured baPWV on both the right and left sides, and the average values in each individual were subjected to statistical analysis. The cut-off point of baPWV was using 1400 cm/s and 1800 cm/s, its definition of vascular failure^2^.

MR-proADM plasma concentrations were measured by an automated KRYPTOR analyzer, using a time-resolved amplified cryptate emission (TRACE) technology assay (Thermo Fisher Diagnostics K.K., Japan), according to the manufacturer’s instruction manuals^14^. Briefly, TRACE is measured by a sandwich immunoassay, wherein the Europium Cryptate, and Cyanine 5 are bound to specific antibodies. This homogeneous assay uses time-resolved measurements and spectral selection to eliminate the background signal of the media and select the specific signal. The plasma sampling was from an antecubital vein.

### Statistical analysis

Continuous variables are expressed as means ± standard deviation (SD) and categorical data are expressed as sums and percentages. Since MR-proADM shows gender differences, we analyzed by gender. Inter-group comparisons were performed using Welch’s t-tests for continuous variables. Tests for linear trends (e.g., P trend tests) were conducted by the sum of four cardiometabolic diseases (obesity, hypertension, dyslipidemia, and diabetes). Spearman’s rank correlation analysis was performed to assess the relationship between the baPWV and other variables, including anthropometric and blood chemistry data, because MR-proADM does not show a normal distribution. Multiple regression analysis was performed to assess the association of MR-proADM and hsCRP, a classical cardiovascular biomarker, with baPWV, adjusting for other variables. The diagnostic performance (accuracy) of MR-proADM with regard to the index of vascular failure was tested with the help of ROC curve analysis in the models. For comparative purposes, the corresponding AUC were calculated and reported. Using G*Power version 3.1.9.6 (http://www.gpower.hhu.de), we verified that the sample size was sufficient. A sample size was calculated with a 0.80 power at the 0.05 alpha level. All statistical analyses were performed using JMP version 13 software (SAS Institute Inc., Cary, NC, USA), and *p* < 0.05 was considered statistically significant.

## Supplementary Information

Supplementary Information.
