# Author Correction: Mid-regional pro-adrenomedullin is a novel biomarker for arterial stiffness as the criterion for vascular failure in a cross-sectional study

**DOI:** 10.1038/s41598-021-96984-3

**Published:** 2021-08-30

**Authors:** Teruhide Koyama, Nagato Kuriyama, Yosuke Suzuki, Satoshi Saito, Ryota Tanaka, Motoshi Iwao, Megumu Tanaka, Takakuni Maki, Hiroki Itoh, Masafumi Ihara, Takayuki Shindo, Ritei Uehara

**Affiliations:** 1 Department of Epidemiology for Community Health and Medicine, Kyoto Prefectural University of Medicine, Kyoto, Japan; 2 Department of Medication Use Analysis and Clinical Research, Meiji Pharmaceutical University, Kiyose, Japan; 3 Department of Clinical Pharmacy, Oita University Hospital, Oita, Japan; 4 Department of Neurology, National Cerebral and Cardiovascular Center, Suita, Japan; 5 Department of Cardiovascular Research, Shinshu University Graduate School of Medicine, Matsumoto, Japan; 6 Department of Life Innovation, Institute for Biomedical Sciences, Interdisciplinary Cluster for Cutting Edge Research, Shinshu University, Matsumoto, Japan; 7 Department of Neurology, Graduate School of Medicine, Kyoto University, Kyoto, Japan

Correction to: *Scientific Reports* 10.1038/s41598-020-79525-2, published online 11 January 2021

The original version of this Article contained errors in Tables 3 and 4.

“Low-density lipoprotein cholesterol” was incorrectly given as “Low-sensitivity C-reactive protein”.

“High-density lipoprotein cholesterol” was incorrectly given as “High-sensitivity C-reactive protein”.

The incorrect and correct values appear below:

Table 3

Incorrect:

**Table:** 

	Men (n = 702)		Women (n = 1467)	
	Coefficient	*p*-value	Coefficient	*p*-value
Low-sensitivity C-reactive protein	− 0.084	**0.026**	0.112	**< 0.001**
High-sensitivity C-reactive protein	− 0.025	0.505	− 0.105	**< 0.001**

Correct:

**Table:** 

	Men (n = 702)		Women (n = 1467)	
	Coefficient	*p*-value	Coefficient	*p*-value
Low-density lipoprotein cholesterol	− 0.084	**0.026**	0.112	**< 0.001**
High-density lipoprotein cholesterol	− 0.025	0.505	− 0.105	**< 0.001**

Table 4

Incorrect:

**Table:** 

	Men (n = 702)		Women (n = 1467)	
	beta	*p*-value	beta	*p*-value
High-sensitivity C-reactive protein	0.024	0.417	− 0.020	0.257
Low-sensitivity C-reactive protein	− 0.035	0.174	0.007	0.660

Correct:

**Table:** 

	Men (n = 702)		Women (n = 1467)	
	beta	*p*-value	beta	*p*-value
High-density lipoprotein cholesterol	0.024	0.417	− 0.020	0.257
Low-density lipoprotein cholesterol	− 0.035	0.174	0.007	0.660

The original Article has been corrected.

