# Supplementary material for: Mid-regional pro-adrenomedullin is a novel biomarker for arterial stiffness as the criterion for vascular failure in a cross-sectional study
Source: Sci Rep. 2021 Jan 11;11:305. doi: 10.1038/s41598-020-79525-2 (PMC7801498; doi:10.1038/s41598-020-79525-2)
Supplement: Supplementary file 1 — Supplementary Information. [file 41598_2020_79525_MOESM1_ESM.docx]

**Mid-regional pro-adrenomedullin is a novel biomarker for arterial stiffness as the criterion for vascular failure in a cross-sectional study**

Teruhide Koyama^a*^, Nagato Kuriyama^a^, Yosuke Suzuki^b,C^, Satoshi Saito^d^, Ryota Tanaka^c^, Motoshi Iwao^c^, Megumu Tanaka^e, f^, Takakuni Maki^g^, Hiroki Itoh^c^, Masafumi Ihara^d^, Takayuki Shindo^e, f^, Ritei Uehara^a^

^a^Department of Epidemiology for Community Health and Medicine, Kyoto Prefectural University of Medicine, Kyoto, Japan

^b^Department of Medication Use Analysis and Clinical Research, Meiji Pharmaceutical University, Tokto, Japan

^c^Department of Clinical Pharmacy, Oita University Hospital, Oita, Japan

^d^Department of Neurology, National Cerebral and Cardiovascular Center, Osaka, Japan

^e^Department of Cardiovascular Research, Shinshu University Graduate School of Medicine, Nagano, Japan

^f^Department of Life Innovation, Institute for Biomedical Sciences, Interdisciplinary Cluster for Cutting Edge Research, Shinshu University, Nagano, Japan

^g^Department of Neurology, Graduate School of Medicine, Kyoto University, Kyoto, Japan

***Corresponding author:** Teruhide Koyama

Department of Epidemiology for Community Health and Medicine, Kyoto Prefectural University of Medicine

465 Kajii-cho, Kamigyo-ku, Kyoto 602-8566, Japan

E-mail: tkoyama@koto.kpu-m.ac.jp

Tel: +81-75-251-5789

Fax: +81-75-251-5799

| Supplemental Table 1. The means of mid-regional pro-adrenomedullin according to the result of the sum of four cardiometabolic diseases (obesity, hypertension, dyslipidemia, and diabetes) | | | | | | | |
| --- | --- | --- | --- | --- | --- | --- | --- |
| Number of cardiometabolic diseases |  | Men (n = 702) | |  |  | Women (n = 1,467) | |
|  |  | Mean ± SD | trend p-value |  |  | Mean ± SD | trend p-value |
| None | n = 157 | 0.423 ± 0.075 | **0.001** |  | n = 588 | 0.385 ± 0.070 | **0.001** |
| One | n = 255 | 0.463 ± 0.087 |  |  | n = 520 | 0.414 ± 0.075 |  |
| Two | n = 182 | 0.490 ± 0.112 |  |  | n = 259 | 0.448 ± 0.088 |  |
| Three | n = 91 | 0.506 ± 0.109 |  |  | n = 87 | 0.473 ± 0.079 |  |
| Four | n = 17 | 0.506 ± 0.112 |  |  | n = 13 | 0.538 ± 0.154 |  |
| Bold style represents P < 0.05. | | |  |  |  |  |  |
| Analysis were performed by tests for linear trends | | | | |  |  |  |
